# Human Cerebrospinal Fluid Fatty Acid Levels Differ between Supernatant Fluid and Brain-Derived Nanoparticle Fractions, and Are Altered in Alzheimer's Disease

**DOI:** 10.1371/journal.pone.0100519

**Published:** 2014-06-23

**Authors:** Alfred N. Fonteh, Matthew Cipolla, Jiarong Chiang, Xianghong Arakaki, Michael G. Harrington

**Affiliations:** Molecular Neurology Program, Huntington Medical Research Institutes, Pasadena, California, United States of America; Louisiana State University Health Sciences Center, United States of America

## Abstract

**Background:**

Although saturated (SAFA), monounsaturated (MUFA), and polyunsaturated (PUFA) fatty acids are important structural components of neuronal membranes and precursors of signaling molecules, knowledge of their metabolism in Alzheimer's disease (AD) is limited. Based on recent discovery that lipids in cerebrospinal fluid (CSF) are distributed in both brain-derived nanoparticles (NP) and supernatant fluid (SF), we hypothesized that fatty acid (FA) abundance and distribution into these compartments is altered in early AD pathology.

**Methodology and Findings:**

We assayed the FA composition and abundance in CSF fractions from cognitively healthy (CH), mild cognitive impairment (MCI), and AD study participants using gas chromatography - mass spectrometry. In the SF fraction, concentration of docosahexaenoic acid [DHA, (C22:6n-3)] was less in AD compared with CH, while alpha linolenic acid [α-LNA, (C18:3n-3)] was lower in MCI compared with CH. In the NP fraction, levels of SAFAs (C15:0, C16:0) and a MUFA (C15:1) differentiated CH from MCI, while two MUFAs (C15:1, C19:1) and four PUFAs (C20:2n-6, C20:3n-3, C22:4n-6, C22:5n-3) were higher in AD compared with CH. Levels of even-chain free SAFA and total free FA levels were higher in AD, levels of odd-chain free SAFAs, MUFAs, n-3 PUFAs, and total PUFA, were lower in AD compared with CH. Free n-6 PUFA levels were similar in all three groups.

**Conclusions and Significance:**

FA metabolism is compartmentalized differently in NP versus SF fractions of CSF, and altered FA levels reflect the importance of abnormal metabolism and oxidative pathways in AD. Depleted DHA in CSF fractions in AD is consistent with the importance of n-3 PUFAs in cognitive function, and suggests that disturbed PUFA metabolism contributes to AD pathology. This study of FA levels in CSF fractions from different cognitive stages shows potential AD biomarkers, and provides further insight into cell membrane dysfunctions, including mechanisms leading to amyloid production.

## Introduction

Specific mutations that inevitably lead to familial Alzheimer's disease (AD) have been identified [1] but most common late-onset AD is a complex trait. Risk factors have been identified [2], but none are necessary and sufficient to cause AD. Early AD pathology, regardless of genetic underpinnings, is characterized by extracellular β–amyloid, predominantly amyloid_1-42_ (Aβ), deposition [3], and intracellular neurofibrillary tangles with hyperphosphorylated tau protein [4]. Since Aβ derives from the transmembrane amyloid precursor protein (APP) [5], [6], the need to understand the mechanisms that link these multiple risk factors to the Aβ cascade is the justification for characterizing the APP membrane lipid environment in AD.

Post-mitotic neurons are especially vulnerable in aging [7], and membrane lipids are more susceptible over time to peroxidative damage [7]. Compared to other organs, human brain is substantially enriched with lipids, constituting roughly half of the dry mass [8], [9]. Though the major classes of brain lipids have been identified, the majority of the lipid molecular species remain uncharacterized. Thus, a major challenge to understanding the origins of Aβ formation is further characterization of the normal brain versus AD brain lipid environments.

We recently described brain-derived, membrane-rich nanoparticles in cerebrospinal fluid (CSF) [10] that provide a fresh and accessible alternative to brain tissue. These lipid-rich, brain-derived nanoparticles (NP) are present in CSF of older adults, ranging from healthy volunteers to those with varied neurological disorders [10]. The NPs are abundant, heterogeneous, and include typical synaptic and large dense core vesicles [10]. Liquid chromatography, mass spectrometry analyses revealed that NPs have different protein and glycerophospholipid composition compared with the supernatant fluid (SF) fraction of CSF [11]. Others have reported that microRNAs are present in NPs [12]. Based on these findings, we proposed that NPs derived from brain tissues are physiologically distinct from the SF fraction [10].

**Figure 1:**
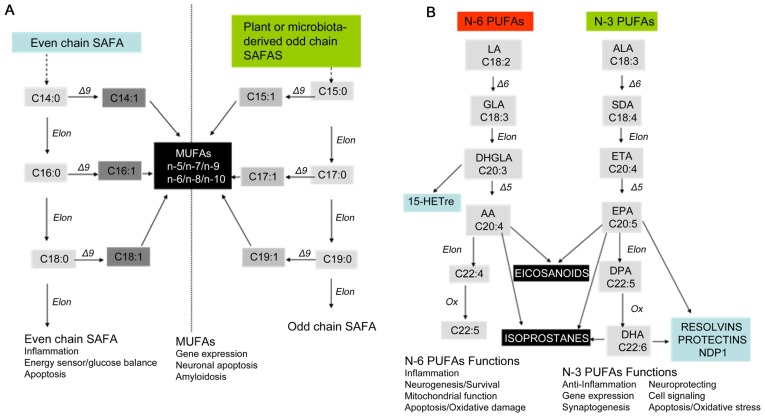
Scheme illustrating fatty acid biosynthesis, metabolism, and function. A) We detected several even-chain saturated fatty acids (SAFAs) in CSF fractions that may be formed by elongase activity that adds two successive carbons during fatty acid synthesis. These SAFAs may be converted by delta 9 desaturase to mono-unsaturated fatty acids (MUFAs). The NP fractions of CSF are also enriched with odd-chain SAFAs and odd-chain MUFAs likely derived from plants or microbiota. SAFA and MUFA levels are differentially altered in cognitive groups indicating disease progression when CH>MCI>AD, or a different pathological mechanism when the profiles are different. B) Polyunsaturated fatty acids (PUFA) are derived from the successive elongation and desaturation of two essential fatty acids: linoleic acid (LA, 18:2n-6) is the precursor of the n-6 PUFA family while alpha linolenic acid (LNA, 18:3n-3) is the precursor of the n-3 PUFA family. Enzymes or non-enzymatic oxidation of these PUFAs form inflammatory eicosanoids and isoprostanes, respectively. The major n-3 PUFAs (EPA, DHA) are metabolized to anti-inflammatory molecules. PUFA levels are differentially altered in cognitive groups suggesting disease progression when CH>MCI>AD, or a different pathological mechanism when the profiles are different.

Of the many lipids that are damaged and accumulate with age, fatty acids (FA) are major components of membranes and are thus important in brain structure and function [13]. FAs are carboxylic acid with aliphatic tails that are major components of glycerophospholipids, sphingolipids, cholesterol esters, and glycerolipids [14], [15]. The aliphatic tails of FAs are composed of different carbon lengths that may be either saturated (SAFA), monounsaturated (MUFA), or polyunsaturated (PUFA). In eukaryotic cells, the most common fatty acyls have even-numbered carbons, although substantial levels of odd-numbered carbon are found in sphingolipids. Using acetyl-CoA as a substrate, FA synthase generates SAFAs that may be desaturated by delta-9 desaturase to form MUFAs (Fig. 1A). No known mechanism exists for the formation of PUFAs, which can be obtained only in the diet (Fig. 1B). Depending on the location of the double bonds, PUFAs are classified as n-3 (omega-3) or n-6 (omega-6). There is some evidence that dietary supplementation with n-3 PUFAs, docosahexaenoic acid (DHA, 22:6n-3), and eicosapentaenoic acid (EPA, C20:5n-3) can enhance memory and cognitive function [16], [17], though this treatment remains somewhat unproven [18].

The biological and physical properties of FAs suggest they impact AD at several levels. SAFAs in cholesterol esters and sphingolipids are components of lipid rafts while dendrites are enriched with PUFAs [19]–[21]. Different compositions of FAs dictate the physical properties of membrane lipids for insulation, the blood-brain-barrier, and the environment for ion channel and receptor functions [22]. FAs are also precursors of signaling molecules such as eicosanoids, endocannabinoids, neuroprotectin, isoprostanes (Fig. 1B) [23], and specialized pro-resolving mediators [24]. Although all PUFAs are important in neuronal functions, high levels of n-6 are generally pro-inflammatory while n-3 fatty acids are generally beneficial. Eicosanoids and isoprostanes generated by enzymatic or non-enzymatic modification of PUFAs are inflammatory, and are implicated in AD pathogenesis [25], [26]. Endocannabinoids and specialized pro-resolving mediators, also generated from PUFAs, are involved in brain plasticity and may help resolve oxidative damage [27]. In addition to providing a milieu for membrane bound proteins, unmodified FAs influence protein function through post-translational modification. One of these, palmitoylation, modifies as much as ten percent of amyloid precursor protein (APP) that is differentially processed to form neurotoxic Aβ[28].

Our aim was to test the hypothesis that FA metabolism in AD or mild cognitive impairment (MCI) is altered compared to cognitively healthy (CH) study participants and that details of the changes would be revealed by study of the NP and SF fractions of CSF. Here we provide the first report of significant changes in SAFAs, MUFAs and PUFAs in the NP fraction from AD and MCI CSF, compared to CH participants, along with cognitive stage-dependent depletion of DHA (22:6n-3) in CSF fractions. Together, these data point to a dysregulation of FA metabolism in CSF fractions that will require further analyses to unravel the role of altered FAs in normal brain, MCI, and AD.

## Materials and Methods

### Ethics statement and diagnosis of study participants

The Institutional Review Board of the Huntington Memorial Hospital, Pasadena, CA, approved the protocol and consent forms for this study (HMH-99-09) and all study participants gave written, informed consent. Participants between 70 and 100 years were recruited. The medical and neuropsychological diagnostic process has been described [29]. Participants were included (Table 1) if they were classified as CH, MCI [30], or with clinically probable AD [31]. APOE genotyping was performed from peripheral blood lymphocytes [32].

**Table 1:** Demographic data, APOE genotype, and CSF levels of Aβ_1-42_ and tau for study participants.

Parameters	CH (Total)	MCI	AD
Total N (with CSF)	70	40	29
Age [mean (SD)]	77.21(6.77)	77.00(6.97)	77.41(9.57)
Female [N (%)]	43(61%)	24(60%)	16(55%)
Years of Education[mean (SD)]	16.34(2.28)	15.45(2.44)	13.89(4.19)**
Race Category – White [N (%)]	68(97%)	39(98%)	24(83%)
Ethnicity - Hispanic or Latino [N (%)]	4(6%)	1(3%)	3(10%)
Homozygous APOE ε4 [N (%)]	2(3%)	3(8%)	3(10%)
Heterozygous APOE ε4 [N (%)]	37(54%)	20(50%)	19(66%)
APOE ε4 Non-Carrier [N (%)]	30(43%)	17(43%)	7(24%)
CSF Aβ_1_-_42_ [mean (SD)]	722.22(299.17)	754.33(268.31)	506.00(230.73)**
CSF Total Tau [mean (SD)]	273.03(149.26)	265.44(168.21)	472.63(221.50)**

** *p<0.05* ANOVA with Dunnett's Multiple Comparison Test.

### CSF collection, total protein, Aβ_42_, and tau measures

After an overnight fast, lumbar puncture was used to collect CSF, between 8:00 am and 10:00 am, which was immediately examined for cell and total protein content. Living and dead cells were counted in a hemocytometer after trypan blue staining. Total protein concentrations were quantified using the fluorescent Quant-iT protein assay kit (Invitrogen/Molecular Probes, Eugene, OR) with bovine serum albumin (0–500 ng/ml) as a standard. CSF was stored in 1 mL aliquots (polypropylene cryo vials, # V9380-100EA, Sigma-Aldrich) at −80°C until thawed for Aβ_1-42_ and Tau assays using a sandwich enzyme-linked immunosorbent assay kit (Innotest β-amyloid_(1-42)_ and Innotest hTAU-Ag, Innogenetics, Gent, Belgium) according to the manufacturer's protocol. All assays were performed in the same week using CSF aliquots after a single thaw.

### Materials

HPLC grade water, chloroform, methanol, formic acid, and anhydrous acetonitrile were purchased from VWR (West Chester, PA). Hydrochloric acid and butylated hydroxytoluene (BHT) were purchased from Sigma (St Louis, MO). Deuterated fatty acid standards (Decanoic Acid-d_19_, Hexadecanoic Acid-d_4_, Oleic Acid-d_17_, Linoleic Acid-d_4_, α-Linolenic Acid-d_14_, Arachidonic Acid-d_8_, Eicosanoic Acid-d_3_, Eicosapentaenoic Acid-d_5_, Docosanoic Acid-d_43_, and Docosahexaenoic Acid-d_5_) were purchased from Avanti Polar Lipids (Alabaster, AL). Non-deuterated standard containing a mixture of 50 free fatty acids was purchased from NuChek Prep (Elysian, MN). Derivatizing reagents Pentafluorobenzyl Bromide (PFBBr) was purchased from Thermo Scientific (Bellafonte, PA) and 99.5% redistilled NN-diisopropylethanolamine (DIPE) from Sigma Aldrich.

### CSF fractionation and fatty acid extraction

CSF (4 mL) was fractionated into a supernatant fluid (SF) or nanoparticle (NP) fractions by centrifugations as previously described [10]. The SF fraction was stored at −80°C and the PBS-washed NP pellet fraction was re-suspended in 25–200 µL of PBS. We added deuterated fatty acid standards to each fraction before fatty acid extraction using a modified Bligh & Dyer procedure [33]. Briefly, a mixture containing 100 ng of each deuterated fatty acid internal standard was added to the NP fraction. After acidification using formic acid (0.9%, 1 mL), we added 2 mL CH_3_OH containing 0.1 mg/mL BHT, and 1 mL CHCl_3_. Drops of CH_3_OH containing 0.1 mg/mL BHT were added if necessary to yield a monophasic clear solution. The solution was vortexed for 5 min and allowed to stand at room temperature for 20 min. To effect phase separation, we added 1 mL NaCl (1 M) and 2 mL CHCl_3_ to each tube. After vortexing (5 min) and centrifugation (1500 RPM, 5 min, 25°C), we collected the lipid-rich lower organic phase using glass pipettes. The top aqueous layer was re-extracted using 2 mL CHCl_3_ and the combined extract dried under a stream of N_2_. The lipids were then dissolved in a 0.5 mL CHCl_3_:CH_3_OH solution (1∶1 v/v, containing 0.5 mg/mL BHT) and stored at −40°C. To determine the fatty acid content, 0.25 mL of the NP extract was subjected to acid hydrolysis and GC/MS as described below.

For the SF fraction, we spiked 1 mL with 100 ng of each deuterated internal standard followed by acidification with formic acid (0.9%, 3 drops) and lipid extraction as described above. The lipids were then dissolved in 0.5 mL CHCl_3_:CH_3_OH solution (1∶1, v/v) containing 0.5 mg/mL BHT. This solution was vortexed for 5 min at room temperature and stored at -40°C. A portion of the SF extract was used to determine the levels of free fatty acids (40%) or subjected to acid hydrolysis to determine the total fatty acid content (20%).

### Acid hydrolysis of extracted lipids

Thawed NP or SF lipid extract aliquots (250 µL) were dried under N_2_ and subjected to hydrolysis as previously described [34]. All solutions (0.5 M HCl in CH_3_CN/H_2_O (9∶1 v/v) were degassed by sonication before addition to borosilicate tubes that were flushed with N_2_ before capping and vortexing for 10 min. The solution was heated for 30 min at 100°C using a heating block with intermittent vortexing every 10 min before organic extraction of the hydrolyzed lipids with CHCl_3_ (2 mL×2). The CHCl_3_ extract was washed using 2 mL NaCl (1 M) by vortexing for 5 min and centrifuging at 1500 RPM for 5 min at 25°C. After removal of the top aqueous layer, 1 mL CH_3_OH containing 0.1 mg/mL BHT was added to the organic extract before drying under a stream of N_2_.

### Derivatization of lipid extracts

Hydrolyzed fatty acids from the NP or SF fractions were converted to pentafluorobenzyl esters using a mixture of PFBBr in acetonitrile solution (1∶19 v/v, 50 µL) and DIPE in acetonitrile solution (1∶9 v/v, 50 µL) for 20 min at 45°C with vortexing every 10 min [35]. The mixture was then dried under N_2_ followed by extraction using 1 mL hexane by vortexing for 5 min and collecting the liquid. To improve the extraction efficiency, the remaining residue was re-extracted with 1 mL hexane by vortexing for 1 min. The hexane extract was dried under N_2_, redissolved in 50 µL dodecane, vortexed for 5 min and sonicated on an Aquasonic water bath for 30 sec before transferring into GC-MS vials.

For determining free fatty acid levels, a thawed SF lipid extract aliquot (300 µL) was dried under N_2_ and derivatized, extracted and transferred into GC vials as described for hydrolyzed CSF fractions.

### GC-MS analyses of derivatized fatty acids

Carboxylate fatty acid ions (m/z) were detected by injecting 1 µL derivatized extracts onto a 7890A GC System coupled to a 7000 MS Triple Quad (Agilent Technologies). Gas chromatography was performed using a Phenomenex Zebron ZB-1MS Capillary GC Column (30 m length, 0.25 mm I.D., 0.50 µm film thickness) heated at 150°C for 1.2 min, ramped to 270°C at 20°C/min and held for 2 min, then ramped to 340°C at 10°C/min and held for 5 min. The temperature of the ion source was 200°C, and the temperature of the quadrupoles was 300°C. Single ion monitoring was used to measure carboxylate ions for deuterated standards and samples. List of carboxylate ions (m/z) for non-deuterated and deuterated fatty acid standards and their retention times are shown in Table S1.

### Data analyses

An Agilent MassHunter Workstation Software was used to analyze GC-MS data. A calibration curve was acquired prior to sample analysis and quality control standards were analyzed periodically throughout the sample runs. Data for compounds that had a consistent QC Accuracy within 80–120% of expected concentrations were accepted and all samples and standards were analyzed in duplicates or triplicates, respectively. Peak integration was automatic for most fatty acids and manual integration was used in selected cases when fatty acid peaks were too broad.

### Statistical analyses

Fatty acid levels in CSF fractions were normalized to the volume of CSF (mean ± SEM, ng/mL), protein content of each fraction (ng fatty acid/ng protein) or as a percent of total fatty acid in each fraction. ANOVA with Tukey's Multiple Comparison tests or the Mann Whitney U tests were performed to determine significant differences in fatty acid levels between CH, MCI, and AD study participants. Spearman's ranked correlation coefficient was used to show the association of free DHA with DHA levels in the NP and SF fractions. All data analyses were performed using GraphPad Prism software (La Jolla, CA) and data considered statistically significant when *P<0.05*.

## Results

### Clinical demographics and AD risk factors

The demographics, AD risk factors, and CSF Aβ_1-42_ and total tau levels are shown in Table 1. The AD group was less educated than other groups as previously reported [29], [36]. The clinical groups were otherwise matched. CSF Aβ_1-42_ was significantly lower while total CSF tau protein was significantly higher in the AD compared with the CH population (Table 1). Thus, the Aβ_1-42_/tau pathology also distinguished our AD population from CH and MCI.

### Fatty acid composition of CSF fractions

Using negative ion NICI GC-MS, we determined whether the fatty acid composition differed between CSF fractions. We detected several SAFAs, MUFAs, and PUFAs in both NP and SF fractions from CH study participants. Of 20 fatty acids detected in the SF fraction, 6 are SAFAs, 6 are MUFAs, and 8 are PUFAs (Table 2). Of 23 fatty acids quantified in the NP fraction, 8 are SAFAs, 5 are MUFAs, and 10 are PUFAs (Table 2). In the SAFA and MUFA subsets, both even-numbered and odd-numbered carbon number fatty acids were detected in both CSF fractions. The total amount of fatty acids in the SF fraction (38,422 ng/ml) was about 22-fold higher than in the NP fraction (1699 ng/ml). The distribution of fatty acid species also differed between these CSF fractions. Apart from C10:0 and C18:0, the proportions of SAFAs were generally higher in the NP fraction compared with the SF fraction (Table 2) with odd chain SAFAs accounting for the biggest difference. The proportions of n-3 PUFAs in NP compared to SF were also generally higher than those of n-6 PUFAs. These differences are more evident when the levels of fatty acids are expressed as a percentage of total fatty acid in each fraction (Table 3). Odd-numbered SAFAs (21X), odd-numbered MUFAs (>4X), and n-3 PUFAs (>5X) were proportionally higher in the NP than in the SF fraction. Even when normalized to the total protein content of each fraction, SAFAs and n-3 PUFAs were enriched in the NP compared to the SF fraction (Table 4). Together, our data show the detection of several fatty acids and differences in the composition of fatty acids between the SF and NP fractions from CSF of CH study participants.

**Table 2:** Fatty acid composition of CSF fractions normalized to CSF volume.

Fatty acids	SF (ng/mL) Mean ± SEM 95% CI), n = 70	NP (ng/mL) Mean ± SEM (95% CI), n = 69,	NP/SF
C10:0	793.5±116.5 (561–1026)	47.4±2.9 (41.6–53.1)	0.06
C11:0	Below LOD	31.3±3.0 (25.3–37.3)	N/A
C14:0	205.4±24.2 (157.1–263.7)	76.6±4.8 (67.1–86.1)	0.37
C14:1	9.6±0.2 (9.2–10.0)	3.4±0.1 (3.3–3.6)	0.36
C15:0	34.6±4.2 (26.3–42.9)	16.4±0.7 (15.0–17.8)	0.47
C15:1	13.2±0.1 (13.1–13.3)	4.6±0.1 (4.4±4.9)	0.35
C16:0	4484±279.4 (3926–5041)	468.8±27.5 (413.9–523.6)	0.11
C16:1	39.5±3.4 (32.7–46.3)	9.2±0.4 (8.4–10.1)	0.23
C17:0	43±11.7 (19.6–66.4)	23.4±1.1 (21.2–25.6)	0.54
C17:1	22.32±1.8 (18.8–25.8)	Below LOD	N/A
C18:0	31,484±8885 (13755–49212)	756.4±78.8 (599–913)	N/A
C18:1	7606±900 (5809–9402)	73.9±5.1 (63.8–84.1)	0.01
C18:2 n-6	715.9±43.9 (628.2–803.5)	18.23±1.5 (15.3–21.1)	0.03
α-C18:3 n-3	22.4±1.4 (18.6–22.2)	Below LOD	N/A
γ-C18:3n-6	894.9±94.5 (706.3–1083)	47.1±4.2 (38.7–55.5)	0.05
C19:0	Below LOD	2.2±0.1 (1.9–2.4)	N/A
C19:1	Below LOD	2.7±0.1 (2.5–2.8)	N/A
C20:1	37.8±5.0 (27.7–47.8)	Below LOD	N/A
C20:2n-6	Below LOD	5.5±0.1 (5.3–5.8)	N/A
D-γ-C20:3 n-6	28.4±3.0 (22.4–34.3)	1.1±0.1 (0.9–1.2)	0.04
C20:3 n-3	34.1±0.8 (32.4–35.8)	11.7±0.3 (11.2–12.3)	0.34
C20:4 n-6	533.8±35.7, (462.7–605)	7.3±0.3 (6.7–7.8)	0.01
C20:5 n-3	18.8±5.6 (7.2–30.40)	1.7±0.2 (1.4–2.1)	0.09
C22:4 n-6	Below LOD	4.0±0.1 (3.8–4.1)	N/A
C22:5 n-3	Below LOD	7.9±0.2 (7.5–8.2)	N/A
C22:6 n-3	263.2±12.2 (239.9-287.5)	57.1±7.2 (42.7–71.5)	0.22

Abbreviations: CI, confidence interval; D-γ-, dihomo gamma; LOD, limit of detection; N/A, not applicable. Below LOD indicates peaks were not detected or the peak profiles and integration did not meet our quality control.

**Table 3:** Fatty acid distribution in CSF fractions.

Fatty Acids	SF% Total (n = 70)	NP% Total (n = 69)	NP/SF
C10:0	2.07	2.81	1.36
C11:0	below LOD	1.84	N/A
C14:0	0.53	4.49	8.40
C14:1	0.02	0.20	8.08
C15:0	0.09	0.97	10.74
C15:1	0.03	0.27	7.93
C16:0	11.67	27.43	2.35
C16:1	0.10	0.54	5.29
C17:0	0.11	1.38	12.32
C17:1	0.06	below LOD	N/A
C18:0	59.87	45.83	0.77
C18:1	18.82	4.35	0.23
C18:2n-6	1.86	1.07	0.58
α-C18:3n-3	0.06	below LOD	N/A
γ-C18:3n-6	2.33	2.76	1.19
C19:0	below LOD	0.13	N/A
C19:1	below LOD	0.16	N/A
C20:1	0.10	below LOD	N/A
C20:2n-6	below LOD	0.33	N/A
C20:3n-3	0.09	0.69	7.77
D-γ-C20:3n-6	0.07	0.06	0.83
C20:4n-6	1.39	0.43	0.31
C20:5n-5	0.03	0.10	3.91
C22:4n-6	below LOD	0.23	N/A
C22:5n-3	below LOD	0.46	N/A
C22:6n-3	0.69	3.47	5.06
Even chain SAFA	74.14	80.56	1.09
Odd chain SAFA	0.20	4.32	21.37
Even chain MUFA	19.05	5.25	0.28
Odd chain MUFA	0.09	0.43	4.63
N-6 PUFA	5.66	4.88	0.86
N-3 PUFA	0.80	4.72	5.91
**Ratios**	**SF Ratios**	**NP Ratios**	**NP/SF**
Even/Odd SAFAs	367.11	18.67	0.05
Even/Odd MUFAs	205.99	12.27	0.06
(N-6)/N-3)	7.08	1.03	0.15

Abbreviations: LOD, limit of detection; D-γ-, dihomo gamma; N/A, not applicable. Below LOD indicates peaks were not detected or the peak profiles and integration did not meet our quality control. Ratios of Even to Odd number carbon fatty acids for SAFAs and MUFAs were calculated from the sum of the percentage of each group in either SF or NP fractions.

**Table 4:** Fatty acid levels normalized to CSF protein content.

Fatty acids	SF ng/ng protein Mean ± SEM (95% CI), n = 70	NP ng/ng protein Mean ± SEM(95% CI), n = 69	NP/SF
C10:0	0.002±0.0025 (0.0003–0.0014)	0.0346±0.072 (0.0281–0.0411)	17
C14:0	0.0005±8E-05 (0.0004–0.0007)	0.0608±0.0604 (0.0463-0.0753	112
C14:1	2E–05±1E-06 (2E-5–3E-05)	0.0026±0.0002 (0.0021–0.0031)	104
C15:0	9E-05±1E-05 (7E-05–0.0001)	0.0123±0.0012 (0.01–0.0146)	132
C15:1	3E-05±1E-06 (3E-05–4E-05)	0.0035±0.0003 (0.0029–0.0041)	102
C16:0	0.0115±0.0009 (0.0098–0.0132)	0.3579±0.0385 (0.281–0.4348)	31
C16:1	0.0001±1E-05 (8E-05–0.0001)	0.0071±0.0009 (0.0052–0.0089)	69
C17:0	0.0001±4E-05 (4E-5–0.0002)	0.0175±0.0019 (0.0138–0.0213)	144
C18:0	0.0889±0.0287 (0.0317–0.1461)	0.5998±0.0959 (0.4085–0.7911)	7
C18:1	0.0196±0.0026 (0.0144–0.0248)	0.0594±0.0112 (0.0371–0.0817)	3
C18:2n-6	0.0017±7E-5 (0.0015–0.0018)	0.015±0.0027 (0.0097–0.0202)	9
α-C18:3n-3	5.8E-5± 5.8E-8 (4.5E-05–6.9E-06)	Below LOD	N/A
γ-C18:3n-6	0.0024±0.0003 (0.0017–0.003)	0.0385±0.0068 (0.025–0.052)	16
D-γ-C20:3n-6	7E-05±9E-06 (5E-05–9E-05)	0.0007±1E-04 (0.0005–0.0009)	10
C20:3n-3	9E-05±5E-06 (8E-05–1E-04)	0.0088±0.0008 (0.0072–0.0104)	99
C20:4n-6	0.0013±7E-05 (0.0011–0.0014)	0.0052±0.0005 (0.0043–0.0062)	4
C20:5n-3	5E-05±2E-05 (1E-05–9E-05)	0.0013±0.0002 (0.0009–0.0018)	26
C22:6n-3	0.0007±4E-05 (0.0006–0.0007)	0.0336±0.0046 (0.0245–0.0428)	50

Abbreviations: D-γ-, dihomo gamma.

### Changes in fatty acid composition

To determine the role of fatty acid metabolism when cognitive function is impaired, we compared the levels of fatty acids detected in CSF fractions from CH participants with CSF fractions from age-matched study participants classified as MCI or AD. Using one-way ANOVA with Tukey's multiple comparison, we compared fatty acid levels in the NP fraction from CH with those from MCI and AD (Table 5).

**Table 5:** Fatty acid composition of brain-derived nanoparticles from CH, MCI, and AD study participants.

Fatty acids	Levels in CH (ng/mL) Mean ± SEM (95% CI) n = 69	Levels in MCI (ng/mL) Mean ± SEM (95% CI) n = 40	Levels in AD (ng/mL) Mean ± SEM (95% CI) n = 29
C10:0	47.4±2.9 (41.6–53.1)	51.5±4.0 (43.3–59.7)	48.5±5.0 (38.4–58.7)
C11:0	31.3±3.0 (25.3–37.3)	32.5±3.5 (25.4–39.6)	36.5±8.8 (18.6–54.5)
C14:0	76.6±4.8 (67.1–86.1)	91.9±6.8 (78.1–105.7)	94.1±9.7 (74.2–114.0)
C14:1	3.4±0.1 (3.3–3.6)	4.0±0.3 (3.4–4.7)	4.0±0.3 (3.3–5–4.6)
C15:0**γ	16.4±0.7 (15.0–17.8)	21.5±1.5 (18.3–24.6)	18.7±1.7 (15.2–22.3)
C15:1**α	4.6±0.1 (4.4±4.9)	4.8±0.2 (4.5±5.1)	5.6±0.4 (4.7±6.5)
C16:0	468.8±27.5 (413.9–523.6)	484±33.6 (416.1–551.9)	566.9±59.8 (444.3–589.4)
C16:1* γ	9.2±0.4 (8.4–10.1)	13.6±2.2 (9.2–18.0)	10.3±0.6 (9.1–11.5)
C17:0**γ	23.4±1.1 (21.2–25.6)	32.2±3.1 (25.9–38.5)	29.0±2.0 (25.0–33.0)
C18:0	756.4±78.8 (599–913)	659.6±110.9 (435.3–883.8)	1119.0±260.8 (583.5–1654.0)
C18:1	73.9±5.1 (63.8–84.1)	83.0±9.2 (64.4–101.6)	68.1±6.0 (55.8–80.3)
C18:2n-6	18.23±1.5 (15.3–21.1)	31.5±9.3 (12.8–50.3)	20.7±4.3 (11.8–29.6)
γ-C18:3n-6	47.1±4.2 (38.7–55.5)	88.2±33.4 (20.7–155.7)	50.2±12.8 (23.9–76.5)
C19:0	2.2±0.1 (1.9–2.4)	2.4±0.2 (2.0–2.9)	2.8±0.3 (2.1–3.4)
C19:1***^αβ^	2.7±0.1 (2.5–2.8)	2.8±0.1 (2.6–3.0)	3.2±0.3 (2.7–3.7)
C20:2n-6* α	5.5±0.1 (5.3–5.8)	5.9±0.2 (5.5–6.2)	6.5±0.5 (5.5–7.4)
D-γ-C20:3n-6	1.1±0.1 (0.9–1.2)	1.3±0.2 (0.9–1.7)	1.0±0.1 (0.9–1.2)
C20:3n-3**α	11.7±0.3 (11.2–12.3)	12.5±0.4 (11.7–13.3)	14.0±1.0 (11.9–16.1)
C20:4n-6	7.3±0.3 (6.7–7.8)	7.2±0.4 (6.4–8.0)	7.6±0.5 (6.7–8.6)
C20:5n-3	1.7±0.2 (1.4–2.1)	2.0±0.3 (1.4–2.6)	1.4±0.4 (0.6–2.2)
C22:4n-6**^αβ^	4.0±0.1 (3.8–4.1)	4.0±0.1 (3.8–4.3)	4.6±0.3 (4.0–5.1)
C22:5n-3**α	7.9±0.2 (7.5–8.2)	8.14±0.3 (7.6–8.7)	9.4±0.7 (7.9–10.9)
C22:6n-3	57.1±7.2 (42.7–71.5)	49.2±9.2 (30.6–67.8)	43.8±6.4 (30.8–56.9)

Abbreviations: CI, confidence interval; D-γ-, Dihomo gamma

* p<0.05, ** p<0.01, ***p<0.005 by ANOVA

α *p<0.5* for CH versus AD;

β *p<0.05* for MCI versus CH;

γ *p<0.05* for MCI versus AD by Tukey's Multiple Comparison Test.

#### NP composition of SAFAs in clinical groups

Of a total of 8 SAFAs quantified in the NP fraction, levels of 7 were higher in MCI than in CH and levels of all 8 were higher in AD compared with CH. One-way ANOVA indicated significant group differences in C15:0 and C17:0 (Table 5) and Tukey's multiple comparison tests showed that CH versus MCI accounted for these group differences.

#### NP composition of MUFAs in clinical groups (Table 5)

Of a total of 5 MUFAs quantified in the NP fraction, levels of all were higher in MCI compared with CH while levels of 4 were higher in AD compared with CH. One way ANOVA indicated differences between clinical groups in C14:1, C15:1, C16:1.C19:1.

#### NP composition of PUFAs in clinical groups (Table 5)

Except for DHA which was lower in MCI compared with CH, levels of all other PUFAs were higher in MCI compared with CH. Similarly, except for C20:3n-6, C20:5n-3, and C22:6n-3, levels of all other PUFAs were higher in AD compared with CH. One-way ANOVA indicated significant group differences in C20:2n-6, C20:3n-3, C22:4n-6, and C22:5n-3. Tukey's multiple comparison tests showed that AD and CH (C20:2n-6, C20:3n-3, C22:4n-6, C22:5n-3), and AD versus MCI (C22:4n-6) accounted for these findings.

### SF fatty acid composition in cognitive groups

We next compared fatty acid levels in the SF fraction from CH with those from MCI and AD (Table 6). Levels of most SAFAs, MUFAs and PUFAs were lower in MCI and AD compared with CH (Table 6). One-way ANOVA indicated significant group differences in α-C18:3n-6 and C22:6n-6. Tukey's multiple comparison tests showed that MCI and CH (α-C18:3n-3), and AD versus CH (C22:6n-3) accounted for these group differences.

**Table 6:** Fatty acid composition of supernatant fluid from MCI and AD study participants.

Fatty acids	Levels in SF (ng/mL) Mean ± SEM 95% CI) n = 70	Levels in MCI (ng/mL) Mean ± SEM 95% CI) n = 39	Levels in AD (ng/mL) Mean ± SEM 95% CI) n = 28
C10:0	793.5±116.5 (561–1026)	645.6±114.6 (413.6–877.6)	506.8±96.9 (307.9–705.6)
C14:0	205.4±24.2 (157.1–263.7)	186.4±15.1 (155.8–217.1)	167.9±13.23 (140.8–195.1)
C14:1	9.6±0.2 (9.2–10.0)	9.47±0.14 (9.18–9.76)	9.2±0.1 (9.1–9.33)
C15:0	34.6±4.2 (26.3–42.9)	35.7±3.7 (27.9–42.7)	26.9±2.9 (20.9–32.8)
C15:1	13.2±0.1 (13.1–13.3)	13.2±0.04 (13.09–13.27)	13.1±0.01 (13.06–13.11)
C16:0	4484±279.4 (3926–5041)	3767.0±224.7 (3313–4222)	3962.0±247.7 (3453–4471)
C16:1	39.5±3.4 (32.7–46.3)	42.1±4.3 (33.4–50.7)	31.7±2.3 (27.0–36.4)
C17:0	43±11.7 (19.6–66.4)	31.9±3.7 (24.4–39.4)	24.4±2.3 (18.3–30.4)
C17:1	22.32±1.8 (18.8–25.8)	21.0±0.5 (19.9–22.1)	19.6±0.4 (18.7–20.5)
C18:0	31,484±8885 (13755–49212)	21770±4160 (13347–30192)	25783±3619 (18357–33209)
C18:1	7606±900 (5809–9402)	6839±1295 (4219–9460)	5737±744.3 (4210–7264)
C18:2n-6	715.9±43.9 (628.2–803.5)	697.00±43.9 (628.2–803.5)	782.9±87.1 (604.3–961.6)
α-C18:3n-3* β	22.4±1.4 (18.6–22.2)	16.3±0.8 (14.8–17.8)	19.2±1.1 (16.9–21.5)
γ-C18:3n-6	894.9±94.5 (706.3–1083)	716.6±64.1 (586.8–846.1)	822.8±88.2 (641.9–1004)
C20:1	37.8±5.0 (27.7–47.8)	33.4±1.1 (31.2–5.6)	28.3±0.9 (26.4–30.2)
D-γ-C20:3n-6	28.4±3.0 (22.4–34.3)	25.5±3.2 (19.1–31.9)	23.3±2.8 (17.7–29)
C20:3n-3	34.1±0.8 (32.4–35.8)	33.3±0.2 (32.9–33.7)	32.7±0.2 (32.2–33.1)
C20:4n-6	533.8±35.7, (462.7–605)	518.0±36.9 (443.3–592.6)	517.3±51.4 (411.8–622.8)
C20:5n-3	18.8±5.6 (7.2–30.40)	10.3±1.8 (6.6–14.0)	6.0±0.8 (4.3–7.64)
C22:6n-3* α	263.2±12.2 (239.9–287.5)	242.6±13.5 (215.4–269.9)	203±13.7 (174.8–231.2)

Abbreviations: CI, confidence interval; D-γ-, Dihomo gamma.

* *p<0.05* by ANOVA.

α *p<0.05* for CH versus AD;

β *p<0.05* for CH versus MCI by Tukey's Multiple Comparison Test.

### Free fatty acid levels in AD

Since we recently showed an increase in PLA_2_ activity and lysophospholipid levels in CSF from AD participants compared with CH/MCI [11], we quantified PLA_2_ products, free fatty acid, to determine if these further reflected stages of AD pathology (Table 7).

**Table 7:** Free fatty acid levels in CSF from CH, MCI, and AD study participants.

Free Fatty acids	Levels in CH (ng/mL) Mean ± SEM (95% CI) n = 69	Levels in MCI (ng/mL) Mean ± SEM (95% CI) n = 38	Levels in AD (ng/mL) Mean ± SEM (95% CI) n = 28
C10:0***α	35.7±1.8 (32.1–39.3)	32.3±2.3 (27.7–36.8)	25.2±2.3 (20.5–29.9)
C11:0***^αβ^	51.3±3.5 (44.2–58.3)	59.6±3.6 (52.3–66.8)	36.7±5.3 (25.8–47.6)
C15:0* α	14.6±0.3 (14.0–15.3)	14.5 0.4 (13.7–15.3)	13.2±0.5 (12.3–14.1)
C16:0**β	271.8±9.0 (253.8–289.8)	237.3±12.1 (212.8–261.8)	295.6±19.0 (256.7–334.5)
C16:1* β	18.6±0.3 (18.1–19.2)	19.6±0.9 (17.9–21.4)	17.4±0.2 (17.0–17.8)
C17:0* γ	10.6±0.5 (9.6–11.6)	8.6±0.6 (7.3–9.9)	10.7±0.6 (9.5–11.8)
C18:0****^αβγ^	211.9±14.2 (183.5–240.2)	152.9±16.7 (119.2–186.7)	282.0±26.4 (227.8–336.1)
C18:1	43.5±3.5 (37.0–50.9)	42.6±4.6 (33.3–51.9)	33.9±2.5 (28.8–39.0)
C18:2n-6	2.5±0.6 (1.3–3.7)	4.4±1.8 (0.6–8.1)	1.8±1.0 (−0.21–3.9)
C20:1	23.7±0.1 (23.6–23.8)	23.9±0.2 (23.5–24.4)	23.7±0.3 (23.0–24.4)
C20:2n-6* ^βγ^	15.5±0.1 (15.4–15.6)	15.9±0.2 (15.5–16.3)	15.4±0.2 (15.1–15.6)
C203n-3	34.4±0.2 (34.0–34.8)	34.4 6± 0.3 (34.1–35.2)	33.7±0.3 (33.1–34.2)
D−γ-C20:3n-6	1.4±0.1 (1.2–1.5)	1.5±0.3 (0.8–2.2)	1.2±0.1 (1.0–1.4)
C20:4n-6	9.6±0.2 (9.1–10.0)	10.3±0.6 (9.0–11.6)	9.4±0.4 (8.6–10.2)
C20:5n-3	1.9±0.4 (1.2–2.7)	1.2±0.4 (0.3–2.1)	1.0±0.2 (0.6–1.5)
C22:1	70.2±0.1 (70.0–70.4)	70.9±0.8 (69.3–72.4	71.8±2.2 (67.6–75.9)
C22:4n-6	8.0±0.04 (7.9–8.1)	8.3±0.3 (7.7–8.8)	8.1±0.1 (7.9–8.3)
C22:5n-3	22.0±0.03 (22.0–22.1)	22.2±0.2 (21.8–22.7	22.0±0.1 (21.9–22.1)
C22:6n-3* ^αβ^	16.8±0.4 (16.0–17.5)	17.1±0.6 (15.9–18.3)	15.3±0.4 (14.4–16.1)

Abbreviations: CI, confidence interval; D-γ-, Dihomo gamma.

* p<0.05, ** p<0.01, ***p<0.005, **** p<0.0001 by ANOVA.

α *p<0.05* for CH versus AD;

β *p<0.05* for MCI versus CH;

γ *p<0.05* for MCI versus AD by Tukey's Multiple Comparison Test and Mann Whitney test for C22:6n-3.

#### Free SAFAs in clinical groups

We detected 6 free SAFAs in the SF fraction and found a decrease in the levels of some in AD compared with CH and in MCI compared with CH (Table 7). Tukey's multiple comparison tests showed that free levels of C17:0 and C18:0 were lower in MCI compared with CH. Free C10:0, C11:0, C15:0, and C18:0 levels were significantly lower in AD compared with CH and levels of C11:0 was lower while C16:0, and C18:0 were higher in MCI compared with AD.

#### Free MUFAs in clinical groups

Of 4 free MUFAs detected in SF, only the levels of C16:1 differed between clinical groups (Table 7). Multiple comparison tests showed that free C16:1 levels were higher in MCI compared with AD.

#### Free PUFAs in clinical groups (Table 7)

9 free PUFAs were detected in the SF fraction. Free C20:2n-6 levels were higher in MCI compared with CH and AD. Free levels of C22:6n-3 were lower in AD compared with CH and MCI.

### Total free SAFAs, MUFAs and PUFAs

**Figure 2:**
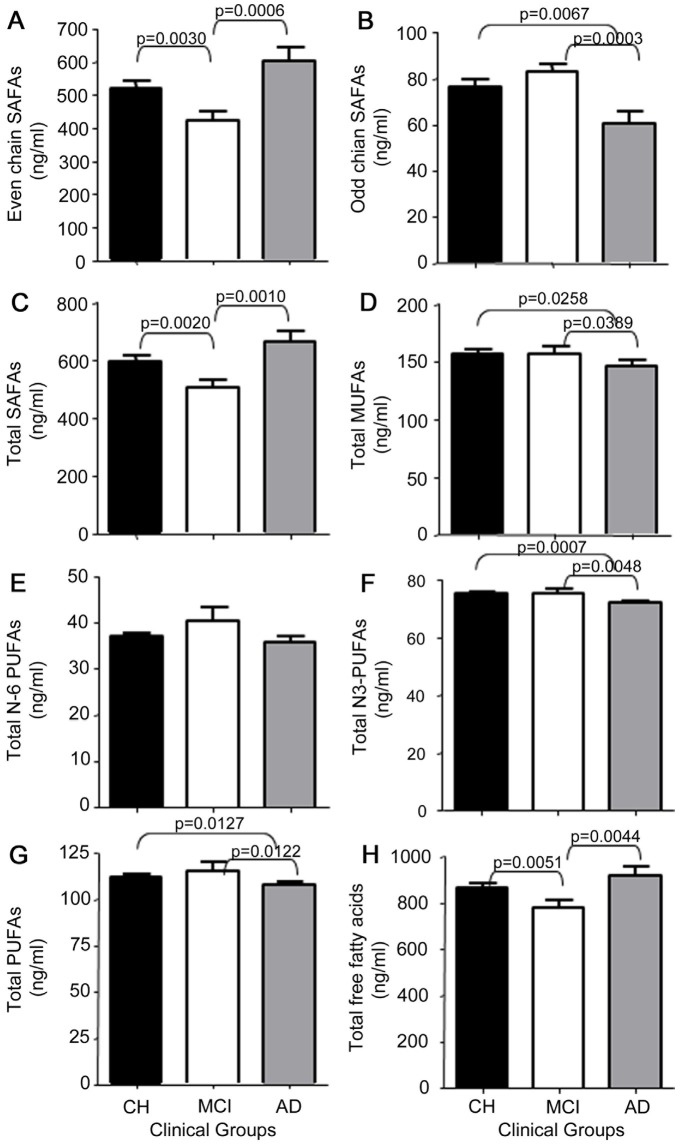
Free fatty acid groups. Free fatty acids were grouped as even-chain SAFAs (A), odd-chain SAFAs (B), total SAFAs (C), total MUFAs (D), n-6 PUFAs (E), n-3 PUFAs (F), total PUFAs (G), and total free fatty acids (H). One-way ANOVA was used to determine group differences and Tukey's multiple comparison test to compare CH (black bar), to MCI (white bar), and AD (gray bar). Data are the mean ± SEM for 68 CH, 38 MCI, and 28 AD study participants with *p* values indicated on brackets.

We next determined total levels of free SAFAs, free MUFAs and free PUFAs in CSF since high SAFA levels have been associated with inflammatory responses that can be attenuated by n-3 PUFA in a dose-dependent manner [37], [38]. Free even-chain SAFA levels were higher in AD than MCI (*P*<0.05, Fig. 2A) and lower in MCI than in CH CSF (*P*<0.05). In contrast, odd-chain SAFA levels were lower in AD compared with CH (*P*<0.05) and MCI (*P*<0.05, Fig. 2B). Total free SAFA levels were higher in AD than MCI and MCI levels were lower than CH (Fig. 2C). Total free MUFA levels were lower in AD than MCI (*P*<0.05, Fig. 2D) and CH (*P*<0.05, Fig. 2D). Whereas free n-6 PUFA levels were similar in all three clinical groups (Fig. 2E), free n-3 PUFA levels were lower in AD compared with CH (*P*<0.05) and MCI (*P*<0.05, Fig. 2F). Total free PUFA were lower in AD compared with CH (*P*<0.05) and MCI (*P*<0.05, Fig. 2G). Despite lower odd- chain SAFA, MUFAs and n-3 PUFAs, total free fatty acid levels in AD were higher than in MCI (*P*<0.05, Fig. 2H), and total free fatty acids in MCI were significantly lower than in CH (*P*<0.05, Fig. 2H).

### Metabolic origin of DHA

**Figure 3:**
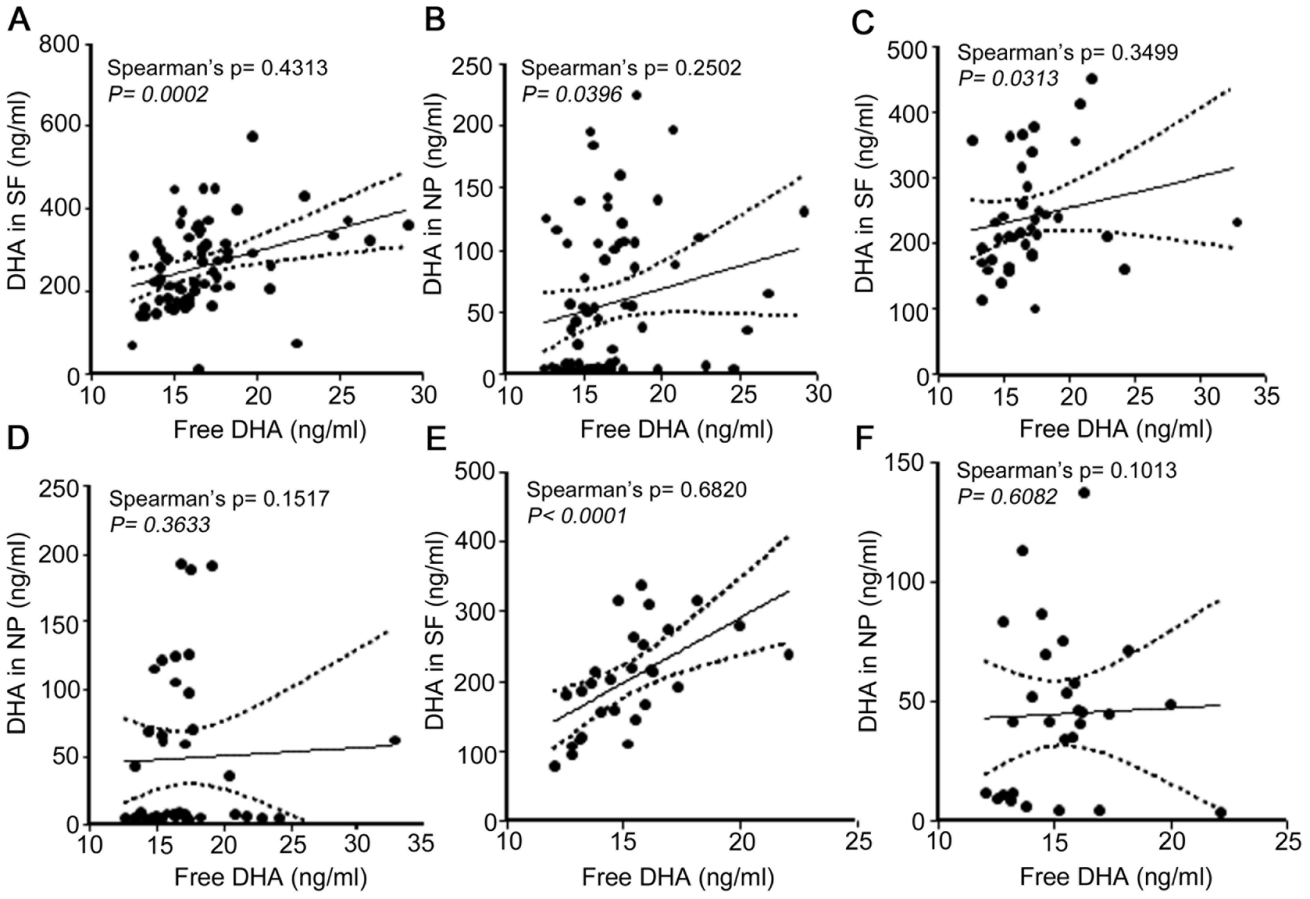
Correlation of DHA levels in clinical groups. Dependence of free DHA on levels in CSF fractions was determined using the Spearman's ranked correlation coefficient (ρ). A) Free DHA versus DHA levels in SF for CH subjects. B) Free DHA versus DHA levels in the NP fraction for CH subjects. C) Free DHA versus DHA levels in SF for MCI subjects. D) Free DHA versus DHA levels in the NP fraction for MCI subjects. E) Free DHA versus DHA levels in SF for AD subjects. F) Free DHA versus DHA levels in the NP fraction for AD subjects.

Examination of fatty acid level changes shows a general decrease in MCI and AD compared to CH for most fatty acids in the SF fraction. In contrast, most fatty acid levels in the NP fraction from MCI (21/23) and AD (19/23) were more abundant than in CH. For free fatty acid levels, 7 out of 19 were lower in MCI than in CH, and 14 out of 19 are lower in AD compared with CH. While this trend holds for many fatty acids, we noticed that C22:6n-3 levels were lower in MCI and AD in both the SF and NP fractions. Further analyses showed that free C22:6n-3 levels in CH correlated more with levels in the SF fraction (Fig. 3A) than with levels in the NP fraction (Fig. 3B). For the MCI subjects, free C22:6n-3 levels correlated with the SF fraction (Fig. 3C) but not with the NP fraction (Fig. 3D). Similarly in AD subjects, free C22:6n-3 levels correlated only with the SF fraction (Fig. 3E) and not with the NP fraction (Fig. 3F).

## Discussion

We recently characterized two CSF fractions composed of supernatant fluid (SF) and brain-derived, membrane rich nanoparticles (NP) [10]. In this study, we used GC-MS to show differences in the fatty acid composition of these CSF fractions and in levels of several free fatty acids. The SF fraction in cognitively healthy participants had higher levels of fatty acids than the NP fraction of CSF, as well as a different distribution of individual fatty acids in each fraction. Proportions of different SAFAs and MUFAs differed in the NP compared with the SF fraction (Table 2). Considering the distribution of the major PUFA in CSF, the ratio of arachidonic acid (C20:4n-6) in NP compared to SF was lower than EPA (C20:5n-3) or DHA (C22:6n-3), suggesting that n-3 fatty acids are relatively enriched in the NP fraction. When normalized to proteins, we found that the NP fraction was substantially enriched with all fatty acids. These studies indicate that the NP and SF fractions are derived from different brain components

Detection of measurable amounts of odd-numbered chain carbon fatty acids in the CSF fractions is an interesting observation from our study. We are not aware of any previous reports of these fatty acids in CSF. Since it is generally assumed that most fatty acids in eukaryotes are even-numbered chains [14], researchers may not have looked for odd-chain numbered fatty acids. The percentage of total odd-numbered carbon fatty acids in the NP fraction was 21-times more than in the SP fraction. Similarly, the percentage of odd-numbered carbon MUFAs in the NP fraction was over four-fold higher than in the SF fraction. Since sphingolipids are enriched with odd-chain fatty acids and are major components of lipid rafts, a possible source of odd-chain fatty acids in the NP fractions is membrane-linked lipid rafts. Enrichment in the NP fraction also suggests that odd-numbered carbon MUFAs and SAFAs may play important roles in the properties of brain membranes than previously recognized.

### Fatty acid composition differs in a stage-specific manner in MCI and AD (Table 8)

Changes in FA composition may be due to uptake, reacylation, oxidation, or PLA_2_-catalyzed hydrolyses of glycerophospholipids. We recently showed an increase in PLA_2_ activity and lysophospholipid levels in CSF from AD participants compared with CH or MCI [11], which stimulated our interest in quantifying free fatty acid levels in AD study participants. While it is impossible to determine the precise sources of free fatty acid in CSF, the magnitude of changes and any correlation with levels in the SF or NP fractions may indicate a source for any particular fatty acid. For example, the increase in fatty acid levels in the NP fraction concomitant with a decrease in the SF fraction may point to a defect in membrane fatty acid transport, trafficking, or remodeling.

While levels of some fatty acid decreased in the SF fraction or increased in the NP fraction, C22:6n-3 levels were consistently lower when comparing AD or MCI to CH subjects (Table 8). These results are counter-intuitive since the increased PLA_2_ activity and decreased glycerophospholipid content that we previously described in CSF [11] would predict higher free DHA in the CSF from AD study participants. A likely explanation is from other pathways that regulate DHA abundance. Dietary deficiency, decreased uptake into the brain, or increased oxidation may account for lowered DHA levels in CSF fractions. Non-enzymatic oxidation of DHA forms 4-hydroxyhexanal (4-HHE), neuroprostanes, neuroketals, and neurofurans [39]–[42]. In addition, DHA released from membrane lipids by PLA_2_ activity has been shown to be converted by lipoxygenase activity to docosanoids and neuroprotectins [43]. Thus, changes in the expression or activation of enzymes and increased non-enzymatic oxidative processes may alter DHA levels and thus influence AD progression.

**Table 8:** Fatty acids that change significantly between diagnostic groups in CSF fractions.

CSF fraction	MCI<CH	MCI>CH	AD<CH	AD>CH	AD<MCI	AD>MCI
**NP**		C15:0		C15:1		C19:1
		C17:0		C19:1		C22:4n-6
		C16:1		C20:2n-6		
				C20:3n-3		
				C22:4n-6		
				C22:5n-3		
**SF**	α-C18:3n-3		C22:6n-3			
**Free fatty acids**	C17:0	C20:2n-6	C10:0	C18:0	C11:0	C16:0
	C18:0		C15:0		C16:1	C18:0
			C22:6n-3		C22:6n-3	

### Odd chain fatty acids as biomarkers of MCI and AD

The potential importance of odd-chain carbon fatty acids in brain pathology is indicated by the changes observed in MCI and AD CSF. For example, levels of two odd- numbered SAFAs (C15:0, C17:0) were significantly higher in the NP fraction of MCI compared with CH subjects. We also measured increased levels of two odd-numbered MUFAs (C15:1, C19:1) in the NP fraction of AD compared with CH subjects. These data suggest that MCI and early AD are associated with more release and potentially more breakdown of lipid membrane as nanoparticles into the CSF compared with CH brains. These nanoparticles that may originate from degenerative neuronal membranes may be useful as biomarkers that distinguish MCI from CH (C15:0, C17:0) or AD from CH (C15:1, C19:1).

### Free SAFAs and inflammation

Studies using cultured astrocytes show that free long chain SAFAs acting on toll-like receptor 4 stimulate TNFα and IL-6 release [44]. These pro-inflammatory effects of SAFAs were dose-dependently attenuated by free n-3 PUFA [44]. The present study indicates that total free fatty acid levels, likely released from glycerophospholipids by PLA_2_ activity as we reported recently (11), were higher in AD. While even-chain SAFA levels were higher, odd chain SAFAs, MUFAs, and n-3 PUFA levels were lower in AD, suggesting that different metabolic pathways may be responsible for the mobilization of these various fatty acid groups. While MUFAs and PUFAs are subject to non-enzymatic oxidation, odd-chain SAFA lipid pools may not be good substrates or may not be accessible to PLA_2_ enzymes, hence their lower free levels and their accumulation in the NP fraction. Overall, higher levels of SAFAs can be expected to increase inflammatory responses and so may contribute to neurologic dysfunction in AD. Further, decreased levels of the natural inhibitors (n-3 PUFA) of these inflammatory pathways may enhance the negative effects of the increased free SAFAs we report in AD.

### Fatty acids and brain energy

Proteins that bind and transport fatty acids [45], [46] play important roles in neuronal cell development and function, neurocognition, and brain diseases [45]–[50]. Although the nervous system does not require fatty acids for its direct energy requirements, some of these binding proteins are responsible for brain lipid sensing and nervous control of energy balance [51]. Different fatty acids affect binding proteins in different ways and variations in their levels affect neural control of energy and glucose homoeostasis [52]. Many studies have shown that n-3 fatty acids influence neurological health through the interaction of metabolic and plasticity signals [53]–[56]. The decreased free n-3 fatty acids that we report in CSF may similarly influence the energy balance and potentiate pathological phenotypes associated with AD.

### Role of MUFAs in AD pathophysiology

Consumption of a Mediterranean diet consisting of olive oil, nuts, unrefined cereals, fruits and vegetables has been linked with reduced risk of Alzheimer's disease [57]–[59], though dietary effects remain unproven [18]. Olive oil is enriched with triacylglycerols containing as much as 55–83% oleic acid (C18:1, n-9), flavonoid polyphenol antioxidants, and pigments such as chlorophyll, pheophytin, and carotenoids [60]. Oleic acid reduces APP gene expression, and prevents amyloidosis in a rodent AD model [61]. In human forebrain, oleic acid was shown to increase in glycerophospholipid classes during myelination [62]. In our study, oleic acid was the most abundant MUFA in both CSF fractions and accounted for the significant decrease in free MUFA levels we measured in AD compared with CH or MCI study participants. This suggests that the uptake, biosynthesis or metabolism of oleic acid and perhaps myelin turnover is compromised in AD. These findings also suggest that our measurements of oleic acid and other MUFAs in CSF fractions could be tailored in future case-controlled studies to evaluate the role of diets in normal brain aging and AD pathology.

### DHA(C22:6n-3) and AD pathophysiology

In addition to studies linking PUFA metabolism with inflammation, neuronal protection, neuronal differentiation and trafficking, brain plasticity, and control of neurotransmitter release, several studies have linked fatty acid metabolism with neurotoxic Aβ production, degradation or clearance. Higher levels of DHA and EPA slowed hippocampal and overall brain atrophy in humans [63]. In a recent longitudinal study, age-dependent executive decline was shown to be sensitive to plasma n-3 PUFA levels [64]. The decrease in DHA that we measured in CSF fractions suggests that beneficial effects of this PUFA are blunted in AD. Thus, attempts at supplementing or preventing the decrease of brain DHA may protect brain membrane structure and function, and prevent the neurodegenerative process of AD. Some clinical studies show beneficial effects of n-3 PUFA supplementation on MCI and memory protection [16], [17], while other studies have been negative [18]. It is not known whether DHA supplements reach the brain and/or replenish membrane lipid pools but measuring these altered CSF fatty acid levels could be used to determine how different PUFA formulations can affect brain lipid composition.

### Implications of cognitive stage-specific fatty acid differences

The FA composition of the NP and SF fractions in the CH group differs from those in fractions from both the MCI, and the AD groups. Although ours is a cross-sectional study, these changes between the clinical groups provide many new pathological insights: i) FAs that show a change between CH and MCI, with an even greater change in AD suggest the progression of a similar pathological process, such as neuronal loss. Examples include C17:0 and C22:6n-3 in the SF fraction, C20:3n-3 and C20:2n-6 in the NP fraction, and free C20:5n-3. ii) FAs that are less in the MCI compared to the CH group, but are more in the AD compared to the CH group may suggest that the FA is a manifestation of a different pathology in the MCI than in the AD group. Examples of this pattern include C18:0, C18:2n-6, C18:3n-3 in the SF fraction, C18.0 in the NP fraction, and C18:0, C17:0, and total free FAs in the free FA fraction. iii). FAs that are higher in MCI compared to CH while lower in AD compared to CH also suggest a different pathology in the MCI than in the AD group. Examples of this include C18:1, C20:5n-3 in the SF fraction, C15:0, C17:0, C16:1, C18:2n-6, C20:5n-3 in the NP fraction, and C11:0, C18:2n-6, and total odd-chain free FAs.

### Conclusion

Our studies show distinct differences in fatty acid levels in brain-derived nanoparticles and supernatant fluids of CSF that would not have been detected without specific CSF fractionation. Some fatty acids change in MCI, and then change further in the same direction in AD, suggesting a similar underlying pathology. Levels of other fatty acids are quite different between MCI from AD, suggesting processes involving fatty acids differ as disease progresses. Thus, stage-specific changes in fatty acids have potential to distinguish the different clinical phenotypes of MCI and AD.

The decrease in DHA(22:6n-3) levels in all CSF fractions and the accumulation of fatty acids in the NP fractions of AD study participants is of importance because these changes may alter membrane properties, and influence APP processing and clearance of neurotoxic Aβ_1-42_. Decreased DHA (22:6n-3) in the SF fraction or increased levels of SAFAs or MUFAs in the NP fraction may be potential biomarkers of AD. However this cross-sectional study design should be followed with controlled longitudinal studies to firmly establish roles of FAs on cognitive function, AD pathophysiology, and to evaluate any FAs as disease biomarkers or treatment targets.

## Supporting Information

Table S1 List of fatty acid standards, retention times, deuterated standards used for GC-MS quantification.
(DOC)
