# Supplementary material for: Human Cerebrospinal Fluid Fatty Acid Levels Differ between Supernatant Fluid and Brain-Derived Nanoparticle Fractions, and Are Altered in Alzheimer's Disease
Source: PLoS One. 2014 Jun 23;9(6):e100519. doi: 10.1371/journal.pone.0100519 (PMC4067345; doi:10.1371/journal.pone.0100519)
Supplement: Table S1 — List of fatty acid standards, retention times, deuterated standards used for GC-MS quantification. (DOC) [file pone.0100519.s001.doc]

**Table S1**: List of fatty acid standards, retention times, deuterated standards used for GC-MS quantification.

| **Fatty Acids** | **Carbon #, DB** | **GLC-463** | | **Internal Standard** | **Linear**  **Range**  **(ng)** | **R^2^** |
| --- | --- | --- | --- | --- | --- | --- |
|  |  | **m/z** | **RT** |  |  |  |
| Pentanoic acid | C5:0 | 101.0 | 4.103 | C10:0 -d_19_ | 0-100 | 0.886 |
| Hexanoic acid | C6:0 | 115.0 | 4.340 | C10:0 -d_19_ | 0-100 | 0.923 |
| Heptanoic acid | C7:0 | 129.0 | 4.839 | C10:0 -d_19_ | 50-100 | 0.999 |
| Octanoic acid | C8:0 | 143.0 | 5.333 | C10:0 -d_19_ | 0-200 | 0.918 |
| Nonanoate | C9:0 | 157.0 | 5.838 | C10:0 -d_19_ | 0-300 | 0.997 |
| Decanoic-d_19_ Acid | C10:0 - d_19_ | 190.2 | 6.272 | N/A | N/A | N/A |
| Decanoic acid | C10:0 | 171.0 | 6.344 | C10:0 -d_19_ | 0-400 | 0.999 |
| Undecenoic acid | C11:1 | 183.0 | 6.804 | C10:0 -d_19_ | 0-200 | 0.998 |
| Undecanoic acid | C11:0 | 185.0 | 6.842 | C10:0 -d_19_ | 0-200 | 0.997 |
| Dodecenoic acid | C12:1 | 197.0 | 7.310 | C10:0 -d_19_ | 0-400 | 0.991 |
| Dodecanoic acid | C12:0 | 199.1 | 7.348 | C10:0 -d_19_ | 0-300 | 0.980 |
| Tridecenoic acid | C13:1 | 211.0 | 7.846 | C10:0 -d_19_ | 0-200 | 0.995 |
| Tridecanoic | C13:0 | 213.0 | 7.883 | C10:0 -d_19_ | 0-200 | 0.992 |
| Tetradecenoic acid | C14:1 | 225.0 | 8.427 | C16:0 -d_4_ | 0-400 | 0.996 |
| Tetradecanoic acid | C14:0 | 227.1 | 8.495 | C16:0 -d_4_ | 0-400 | 0.998 |
| Pentadecenoic acid | C15:1 | 239.0 | 9.136 | C16:0 -d_4_ | 0-300 | 0.994 |
| Pentadecanoic acid | C15:0 | 241.1 | 9.204 | C16:0 -d_4_ | 0-300 | 0.998 |
| 9-Hexadecenoic acid  (Palmitoleic) | C16:1 | 253.1 | 9.834 | C16:0 -d_4_ | 0-500 | 0.994 |
| Hexadecanoic-7, 7, 8, 8-d_4_ Acid | C16:0-d_4_ | 259.2 | 9.959 | N/A | N/A | N/A |
| Hexadecanoic acid  (Palmitic) | C16:0 | 255.1 | 9.993 | C16:0 -d_4_ | 0-400 | 0.996 |
| 10-Heptadecenoic acid | C17:1 | 267.1 | 10.626 | C16:0 -d_4_ | 0-600 | 0.997 |
| Heptadecanoic | C17:0 | 269.1 | 10.766 | C16:0 -d_4_ | 0-600 | 0.996 |
| 9-12-13 Octadecatrienoic acid (Alpha Linolenic) | α-C18:3n-3 | 277.0 | 11.121 | C18:2 -d_4_ | 0-400 | 0.992 |
| 9-Octadecenoic acid -d_17_ (Oleic Acid d_17_) | C18:1 d_17_ | 298.2 | 11.234 | N/A | N/A | N/A |
| 9-12 Octadecadienoic acid d_4_ (Linoleic acid d_4_) | C18:2 - d_4_ | 283.1 | 11.287 | N/A | N/A | N/A |
| 9-12 Octadecadienoic acid (linoleic acid) | C18:2n-6 | 279.1 | 11.302 | C18:2 -d_4_ | 0-600 | 0.995 |
| 9-Octadecenoic acid (Oleic Acid) | C18:1 | 281.1 | 11.347 | C18:1 -d_17_ | 0-800 | 0.998 |
| 6-9-12 Octadecatrienoic acid (Gamma linolenic acid) | γ-C18:3n-6 | 277.1 | 11.355 | C18:2 -d_4_ | 0-200 | 0.991 |
| Octadecanoic acid | C18:0 | 283.1 | 11.536 | C18:1 -d_17_ | 0-800 | 0.988 |
| 7-Nonadecenoic acid | C19:1 | 295.1 | 12.064 | C20:4 -d_8_ | 0-300 | 0.996 |
| Nonadecanoic acid | C19:0 | 297.1 | 12.298 | C20:4 -d_8_ | 0-200 | 0.980 |
| 5-8-11-14 Eicosatetraenoic acid d_8_ (Arachidonic acid d_8_) | C20:4 –d_8_ | 311.1 | 12.305 | N/A | N/A | N/A |
| 5-8-11-14 Eicosatetraenoic acid (Arachidonic acid) | C20:4n-6 | 303.1 | 12.464 | C20:4 -d_8_ | 0-500 | 0.997 |
| 5-8-11-14-17 Eicosapentaenoic Acid d_5_ (EPA d_5_) | C20:5 -d_5_ | 306.1 | 12.348 | N/A | N/A | N/A |
| 5-8-11-14-17 Eicosapentaenoic Acid (EPA) | C20:5n-3 | 301.0 | 12.513 | C20:5 -d_5_ | 0-600 | 0.996 |
| 8-11-14 Eicosatrienoic acid  (Dihomo-γ-Linolenic acid) | D-γ-C20:3n-6 | 305.1 | 12.660 | C20:5 -d_5_ | 0-300 | 0.998 |
| 5/8-Eicosenoic acid | C20:1 | 309.1 | 12.807 | C20:0 -d_3_ | 0-1200 | 0.992 |
| Eicosadienoic acid | C20:2n-6 | 307.1 | 12.834 | C20:0 -d_3_ | 0-600 | 0.996 |
| 11-Eicosenoic | C20:1 | 309.1 | 12.864 | C20:0 -d_3_ | 0-600 | 0.996 |
| Eicosatrienoic acid | C20:3n-3 | 305.1 | 12.887 | C20:0 -d_3_ | 0-600 | 0.992 |
| Eicosanoic acid -d_3_ | C20:0 -d_3_ | 314.1 | 12.894 | N/A | N/A | N/A |
| C20:0 Arachidate | C20:0 | 311.2 | 13.056 | C20:4 -d_8_ | 0-800 | 0.979 |
| Hen-EPA d_6_ | Hen-EPA d_6_ | 321.1 | 13.237 | N/A | N/A | N/A |
| Docosahexaenoic acid (DHA)-d_5_ | C22:6 -d_5_ | 332.1 | 13.707 | N/A | N/A | N/A |
| Docosahexaenoic acid (DHA) | C22:6n-3 | 327.0 | 13.822 | C22:6 -d_5_ | 0-600 | 0.998 |
| Docosatetraenoic acid | C22:4n-6 | 331.1 | 13.954 | C22:6 -d_5_ | 0-500 | 0.997 |
| Docosapentaenoic acid | C22:5n-3 | 329.1 | 14.003 | C22:6 -d_5_ | 0-600 | 0.996 |
| Docosanoic-d_43_ Acid | C22:0 -d_43_ | 382.5 | 14.215 | N/A | N/A | N/A |
| Docosadienoic acid | C22:2n-6 | 335.1 | 14.279 | C22:0 d_43_ | 0-200 | 0.983 |
| 13-Docosenoic acid | C22:1 | 337.1 | 14.309 | C22:0 d_43_ | 0-1200 | 0.990 |
| Docosatrienoic acid | C22:3n-3 | 333.1 | 14.335 | C22:0 d_43_ | 0-600 | 0.977 |
| Docosanoic acvid | C22:0 | 339.1 | 14.460 | C22:0 d_43_ | 0-1000 | 0.996 |
| 14-Tetracosenoic acid | C24:1 | 365.1 | 15.652 | C22:0 d_43_ | 0-300 | 0.994 |
| Tetracosanoic acid | C24:0 | 367.1 | 15.788 | C22:0 d_43_ | 0-400 | 0.987 |

Abbreviations: DB, double bonds; GLC-463, fatty acid catalogue number from NuChek Prep; RT, retention time; N/A, not applicable
